# Kinetics of Natural Attenuation: Review of the Critical Chemical Conditions and Measurements at Bore Scale

**DOI:** 10.1100/tsw.2002.299

**Published:** 2002-05-16

**Authors:** O. Atteia, M. Franceschi

**Affiliations:** EGID Institute, Bordeaux 3 University, 1 Allée Daguin 33607 Pessac Cedex, France

**Keywords:** natural attenuation, redox, BTEX, modelling, field measurements, degradation rates

## Abstract

This paper describes the chemical conditions that should favour the biodegradation of organic pollutants. Thermodynamic considerations help to define the reaction that can occur under defined chemical conditions. The BTEX (benzene, toluene, ethylbenzene, and xylene) degradation is focused on benzene, as it is the most toxic oil component and also because it has the slowest degradation rate under most field conditions. Several studies on benzene degradation allow the understanding of the basic degradation mechanisms and their importance in field conditions. The use of models is needed to interpret field data when transport, retardation, and degradation occur. A detailed comparison of two existing models shows that the limits imposed by oxygen transport must be simulated precisely to reach correct plumes shapes and dimensions, and that first-order kinetic approaches may be misleading. This analysis led us to develop a technique to measure directly biodegradation in the field. The technique to recirculate water at the borehole scale and the CO_2_ analysis are depicted. First results of biodegradation show that this technique is able to easily detect the degradation of 1 mg/l of hydrocarbons and that, in oxic media, a fast degradation rate of mixed fuel is observed.

## INTRODUCTION

The increasing use of natural attenuation in northern America is the consequence of (1) the extremely long duration and high cost of aquifer rehabilitation by classical methods and (2) the discovery of the existing degradation done by bacteria in a large variety of situations. Natural attenuation is defined as the sum of the processes able to decrease pollutants' concentration at a sampling point in an aquifer. Several physical processes such as dispersion, retardation, and solubility can play a role in natural attenuation. Dispersion may decrease concentration for a pulse of contaminant, but play a small role on continuous injection[1]. Retardation leads to important attenuation when the retardation factor is really high (>20), which is not the case for BTEX. Despite these important effects of physical phenomena, only biodegradation can significantly reduce the total amount of pollutant in an aquifer and allow the pollutant concentration to reach the low concentrations required by regulations.

Intrinsic biodegradation is a complex task to handle because it requires the presence of bacteria and the chemical conditions favouring their growth. This field of research is evolving very quickly and, concerning BTEX, numerous experiments have been done in the laboratory. This conducted to the evidence of degradation under aerobic conditions and a more recent acceptance of the existence of degradation under anoxic conditions, with different electron acceptors[1]. However, as will be detailed below, the experiments do not show converging conclusions for specific substances and chemical conditions. That is why the first part of this paper is focused on a detailed analysis of field data concerning BTEX and particularly benzene degradation.

Important efforts in the field were oriented toward the determination of representative degradation constants, and several values were obtained in well-documented chemical conditions. In a first attempt, the intent to relate degradation rates or plume sizes to classical hydrodynamic parameters failed. This revealed the necessity to understand the chemical structures of contaminated plumes. The second part of the paper tries to give some answers to the complex behaviour of pollutant plumes and detail their evolution by the comparison of different models.

Although the use of numerical models may help to test the role of oxidants on degradation, field data on degradation constants will be more and more necessary. Most of the currently used methods do not directly measure biodegradation and, as will be exposed in the modelling part, rely on hypotheses that are not always verified. That is why the third part of the paper concerns the development of a new tool to measure biodegradation rates in the field, at the borehole scale.

## CHEMISTRY OF BTEX NATURAL ATTENUATION

A detailed analysis of the basic thermodynamic of redox reactions involved in biodegradation is necessary to describe the reactions that can potentially occur. The redox potentials of BTEX show that oxidation is the major degradation reaction. For oxidation, the classical pattern of the use of electron acceptor (EA) in the order of decreasing reaction's free energies ($O_2$, then $NO_3$, $Fe^{III}$, and $SO_4$) for bacteria may apply. Toluene (and sometime ethylbenze and xylene) happens to be degraded by fermentation and thus even under methanogenic conditions. A global comparison of reaction rates obtained from laboratory and field experiments clearly demonstrates that, in field conditions, the feeding of redox reactants is a limiting factor of the reaction kinetics[2].

Degradation of BTEX under field conditions has been largely documented and, except benzene, degradation was shown to occur under almost all redox states. Although bacteria growth requires adaptation periods, these time scales are fairly short, from hours in the laboratory to months in the field[3], and thus it is not the main constraint on degradation. The most resistant contaminant in almost all field studies is benzene. As it is also carcinogenic and thus the most toxic

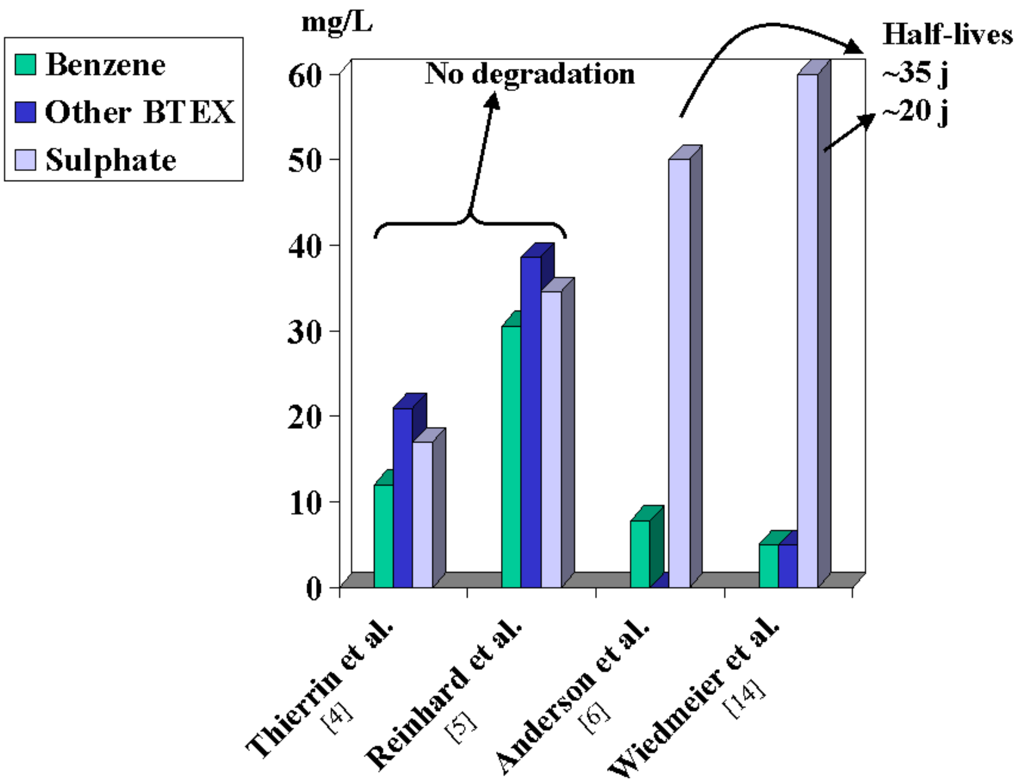

**FIGURE 1.** Benzene degradation under anoxic conditions, sulphate being the major electron acceptor. For each study, the amount of benzene, other BTEX, and sulphate present in water is shown. Benzene biodegradation half-lives were estimated in the field.

compound, our main interest will be targeted toward this molecule. In the laboratory, and a few field studies, the degradation of benzene in oxic conditions has been shown to occur at fast rates. This is justified by the high energy released by the reaction that favours the growth of the catalysing bacteria. The degradation under nitrate-, methane-, or iron-reducing conditions is almost null, while it has been shown to occur under sulphate reducing ones.

Competition between bacteria can play an important role on the relative development of degradation pathways. While two reactions provide similar amounts of energy, there are cases where, according to the temporal variation of chemical conditions one bacteria population develops while others decline[4].

We think that this bacteria competition may explain why some field results show benzene degradation under anaerobic conditions while others do not. Most of the examples concern degradation under sulphate-reducing conditions, as sulphate may exist at high concentrations and also because oxidation by nitrate and iron oxidation is negligible. In Thierrin et al.[5] it is shown, by isotopes measurements, that inside the plume, where sulphate reducing condition prevail, there is no benzene degradation. In strictly sulphate-reducing conditions Reinhard et al.[6] found a negligible degradation of benzene while, on the contrary, Anderson and Lovley[7] demonstrate benzene degradation, with half-lives of 25–35 days. This apparent contradiction can be resolved in the light of other experiments[8] proving that benzene degradation under anoxic conditions occurs only in the absence of any other carbon substrates. Fig. 1 shows that where TEX (toluene, ethybenzene, and xylene) are present in higher amounts than benzene, there is no benzene degradation. On the contrary, benzene can be degraded under sulphate-reducing conditions where it is almost the only hydrocarbon present. This shows that bacteria may degrade benzene but, as it is difficult to degrade, it will be used as the last substrate.

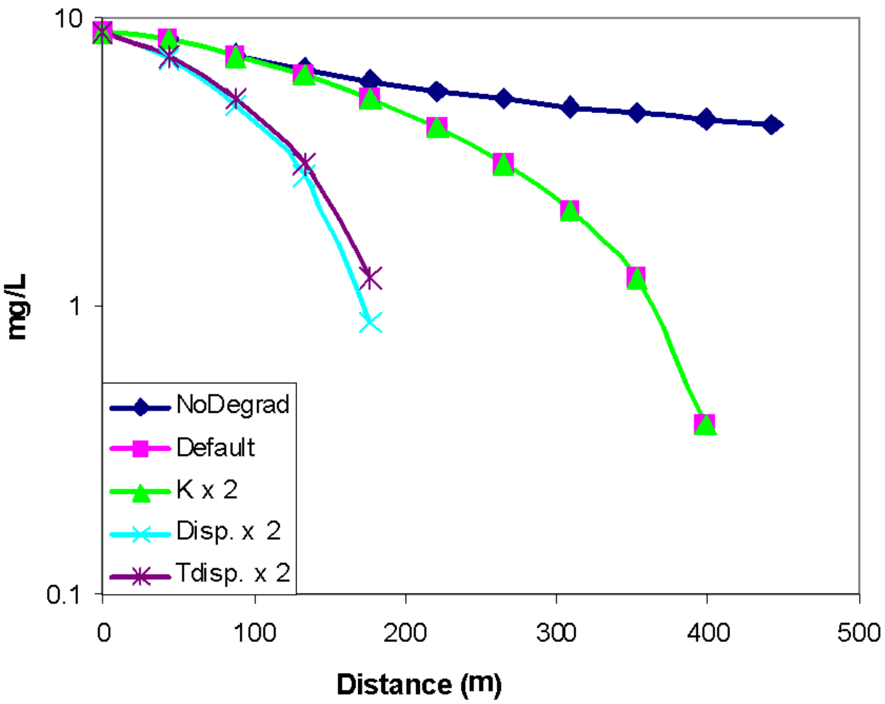

**FIGURE 2.** Tests of BIOSCREEN reply to variations of hydrodynamic characteristics of the aquifer. Default conditions: $K = 8\ 10^{-5}$ m/s, $i = 0.048$, $\omega = 0.25$, $\alpha_L = 8.7$ m, $\alpha_T = \alpha_L/10$, EA: $O_2 = 7.5$ mg/l. NoDegrad : absence of biodegradation, Disp.: dispersivity and T: transverse.

## PLUME DEVELOPMENT AND MODELLING STRATEGIES

Degradation rates of organic substances cannot be assessed easily in the field as concentrations are known only punctually in space, transport properties of the media are estimated, and the history of the contaminant source is not known. Many efforts were made in large field experiments to retrieve precise maps of contaminant concentrations and estimates of biodegradation constants. It is striking to observe that, despite the knowledge of the crucial role of local redox conditions in biodegradation, most of the rate constants extracted from field data are simply first-order relative to the contaminant concentrations. It is therefore necessary to reanalyse the biodegradation facts to provide a more precise canvas to describe field data.

First of all, the complete independence of BTEX plume size on hydrodynamic characteristics of the corresponding aquifers discovered in Newell et al. is surprising[9]. A basic tool to analyse this behaviour is Bioscreen[10], an analytical model of biodegradation working at the plume scale that includes two main options: first-order degradation rate and instantaneous reaction. The concept of instantaneous reaction relies on the fact that most of the degradation rates found in the laboratory are much shorter than the time scales for EA transport in a plume[11].

Results obtained by using Bioscreen with the instantaneous reaction option show that the hydraulic conductivity of the aquifer does not change the plume size at all (Fig. 2). On the contrary, changing dispersivity, or even only transverse dispersivity, has dramatic consequences on plume length. These two effects can be understood in the light of chemical reactions: whatever the ground water velocity is, the most important effect for degradation is mixing between surrounding water containing EA and plume water containing hydrocarbons. Therefore, a model of instantaneous biodegradation can fully explain the independence between BTEX plume size and hydraulic conductivity.

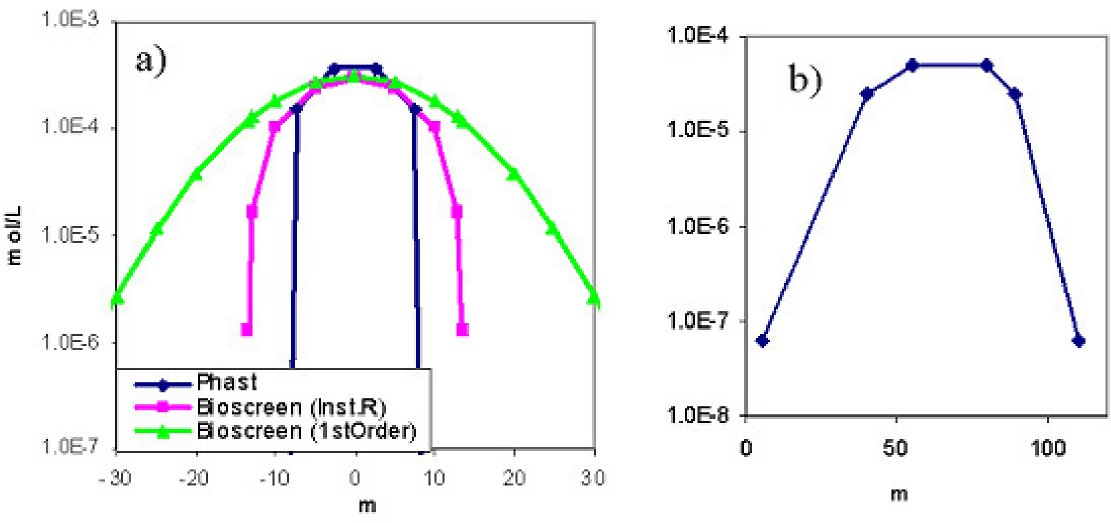

**FIGURE 3.** (A) BIOSCREEN and PHAST simulations of plume transverse profile at 50-m distance from the source, hydrodynamic parameters being equal to the one used in Fig. 2, t = 5 years, Co = 13 mg/l (1 mmol/l of CH compound) in a 10-m wide source. (B) An example of a transverse profile in a BTEX plume: the case of Plattsburgh.

Owing to the crucial role of fast oxic degradation of benzene, the approach of mixing between contaminated and clean water shall be examined in detail. For this purpose, we used two different models:

1. Bioscreen in which the biodegradation is simplified by using superposition, and
2. A much more detailed model able to treat two-dimensional reactive transport: Phast[12], which is a combination of Hst3D[13] for transport and Phreeqc[14] for reaction.

Tested for different size and concentrations of sources, the models compare quite well, giving some confidence in their results as they are based on completely different approaches.

The analysis of transverse profiles is quite demonstrative (Fig. 3a): Phast gives cells containing some mg/l neighbouring cells free of hydrocarbon; Bioscreen "instantaneous reaction" gives plumes very similar to Phast ones, but with slightly smoother borders. On the contrary, a first-order degradation option lead to plumes three times wider. In most of the natural plumes, the transverse profile presents steep concentration gradients. As an example, at Plattsburgh[15], a decrease of approx. three orders of magnitude in BTEX concentration is achieved in 20 m (Fig. 3b). This is impossible to simulate with a first-order decay model.

A longitudinal cut inside the plume also shows that, where biodegradation is active, the plume presents, at its front, a very steep gradient of pollutant concentration (Fig. 4). As for the transverse profiles, this is a consequence of the total oxidation of benzene by oxygen, where the concentration of this last compound is high enough. This leads to concentrations of benzene below regulation standards at much shorter distances in simulations using instantaneous degradation (~380 m) rather than the ones using first-order decay (>600 m). The classical approach of fitting a first-order constant to field data may thus be misleading.

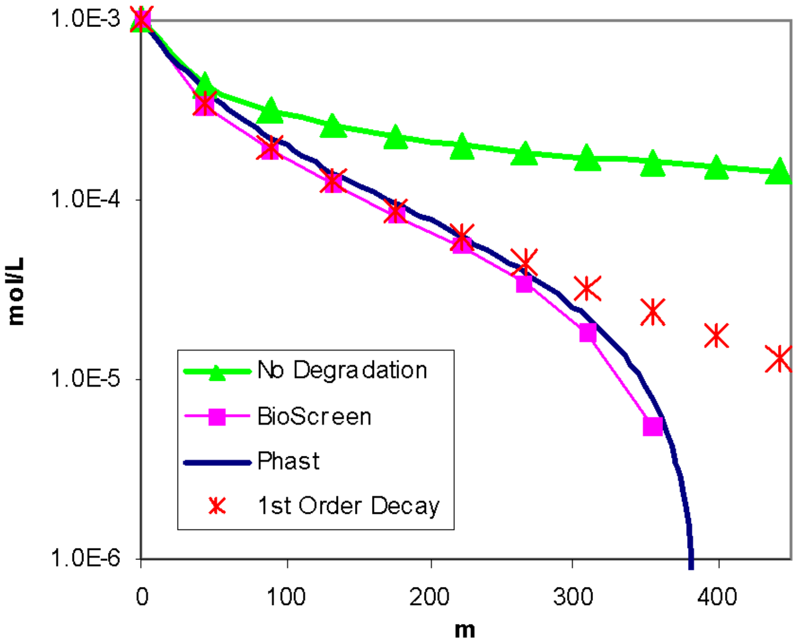

**FIGURE 4.** BIOSCREEN and PHAST simulations of hydrocarbon profiles in the plume centreline, hydrodynamic parameters being equal to the one used in Fig. 2. For first-order degradation, the fitted half-life is equal to 90 days.

## *IN SITU* NATURAL ATTENUATION MEASUREMENTS

As seen in the previous chemistry and plume development sections, field estimates of degradation rates in real conditions are crucial to predict plumes behaviour. That is why we developed an apparatus focused on the measurement of degradation in field conditions. The main objective was to provide a tool to estimate the potential for natural attenuation directly in the field. This apparatus relies on two original processes: (1) the degradation of hydrocarbons is measured by the analysis of $CO_2$ production and (2) the water to be analysed is isolated from ambient air and recirculated. In the field, the $CO_2$ produced by biodegradation may accumulate in water and the difference in concentrations between two boreholes can, in principle, be measured. However, the interpretation of such measurements is always restricted by the heterogeneity of physical and chemical conditions even at the meter scale. By recirculating water in only one borehole, $CO_2$ may accumulate in the water and the investigated volume may remain the same along time. The potential effect of dispersion of $CO_2$ to the surroundings is estimated by the injection of tracers in the flowing system.

The interaction of $CO_2$ with the other dissolved carbonates forms has to be addressed. If calcite, or other carbonates, is absent from the sediment, the concentration of all ions in the following formula will remain constant:

$$\Sigma+ = 2\ Ca^{2+} + 2\ Mg^{2+} + K^{+} + Na^{+} - Cl^{-} - 2\ SO_4^{2-} - NO_3^{-} = \text{Constant} \qquad (1)$$

A solution is electroneutral by definition and, at the groundwater pH, where the major ionic carbonate form is $HCO_3^-$, we obtain:

$$HCO_3^- = \Sigma+ = \text{Constant} \qquad (2)$$

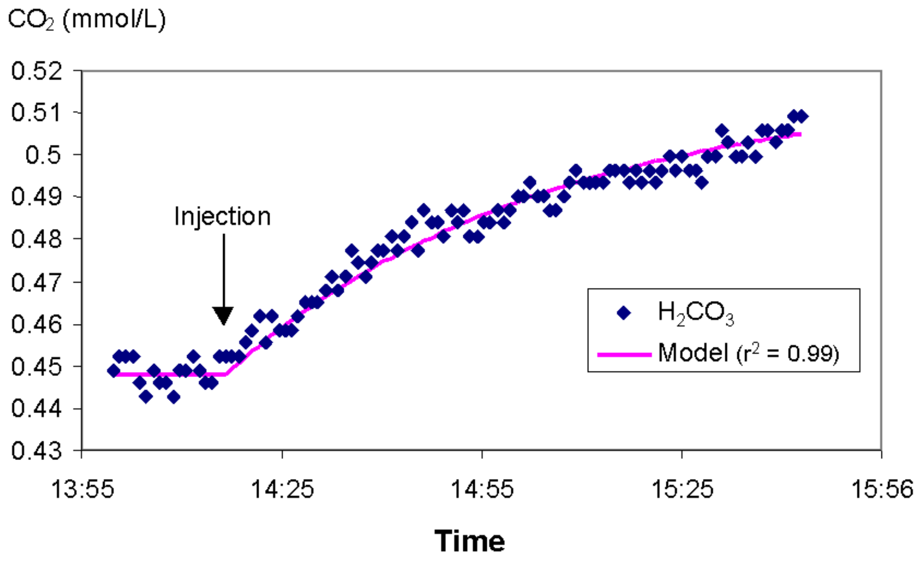

**FIGURE 5.** Degradation of fuel mixture in the experimental apparatus. The equivalent of 1 mg/l (or approx. 0.077 mmol/l of organic C) of fuel was injected. $CO_2$ is given in mmol/l, as measured in gas phase equilibrated with recirculating water, time in hours. The vertical arrow shows the time of injection of the fuel mixture.

Therefore, in such a system, the addition of $CO_2$ will not change the $HCO_3^-$ concentration and, despite a pH change, all the added $CO_2$ will remain in $CO_2/H_2CO_3$ form. The measurements are done in air and we use the Henry's law to convert measured $P_{CO_2}$ to $H_2CO_3$ concentration in water. If the aquifer contains calcite, a different approach should be used, because the addition of $CO_2$ and pH change may lead to calcite dissolution. In that case, the sensitivity of the method is lower by approximately 50%, and results have to be interpreted by geochemical modelling to give real produced $CO_2$.

Presently a laboratory prototype of the apparatus has been realised. It consists of a 200-l barrel full of water-saturated sand that is isolated from ambient air. This pilot has been inoculated by bacteria selected from a site contaminated by hydrocarbons, which where injected in liquid phase, after verification of their degradation ability by ETS (Electron Transport System[16]) measurements. When bacteria were adapted, a few milliliters of fuel were added and an increase in $CO_2$ concentration was observed. Measurements for this fuel injection are shown in Fig. 5 as the evolution of $CO_2$ partial pressure according to time. It is clear that immediately after injection, the $CO_2$ content, which was fairly stable before, starts to rise. We then observe a typical first-order kinetic-type response curve. The apparatus is thus very sensitive to degradation, as this response corresponds to the degradation of only 1 mg/l of fuel dissolved in water. A simple kinetic model fitted to the data shows that an approximate half-life of 1.4 h for degradation of this fuel under oxic conditions can be adopted. This value agrees well with classical values of BTEX mixtures' degradation rates in oxic conditions ranging from hours to days[17]. Further experiments will concern pure substances and various electron acceptors, before field tests.

## CONCLUSION

The analysis of field data on BTEX, and particularly benzene, degradation clearly demonstrates the need for a detailed characterisation of plumes chemistry and of hydrocarbons content. Although benzene degradation may exist under anoxic conditions, it occurs under few specific cases, and thus the role of oxygen feeding to the plume is crucial. To address this mixing process, measurements of transverse dispersion are very useful and shall be advised. First-order kinetic models are not able to reproduce the above phenomena and their results do not agree with field data on transverse profiles. To provide data on actual degradation rates, a simple method is presented; it has been able to measure easily the degradation of 1 mg/l of fuels and is currently under development for field applications.

## ACKNOWLEDGEMENTS

The research on *in situ* natural attenuation measurements is funded by PEA (Pôle Environnement Aquitain). This paper was presented at the CSIC/ESF Workshop, Analysis, Toxicity, and Biodegradation of Organic Pollutants in Groundwater from Contaminated Land, Landfills, and Sediments, Barcelona, Spain, 8–10 November 2001.
